# Validation of ICD-9-CM codes for identification of acetaminophen-related emergency department visits in a large pediatric hospital

**DOI:** 10.1186/1472-6963-13-72

**Published:** 2013-02-21

**Authors:** Sofia de Achaval, Chris Feudtner, Shana Palla, Maria E Suarez-Almazor

**Affiliations:** 1 Department of General Internal Medicine, The University of Texas MD Anderson Cancer Center, 1515 Holcombe Blvd., 77030, Houston, TX, USA; 2 Department of Pediatrics, The Children’s Hospital of Philadelphia, 34th Street and Civic Center Boulevard, 19104, Philadelphia, PA, USA; 3 Department of Biostatistics, The University of Texas MD Anderson Cancer Center, 1515 Holcombe Blvd., 77030, Houston, TX, USA

**Keywords:** Acetaminophen, Overdose, Pediatric, Emergency department, Validity

## Abstract

**Background:**

Acetaminophen overdose is a major concern among the pediatric population. Our objective was to assess the validity of International Classification of Disease (ICD-9-CM) codes for identification of pediatric emergency department (ED) visits resulting from acetaminophen exposure or overdose.

**Methods:**

We conducted a retrospective medical record review of ED visits at Texas Children’s Hospital in Houston, Texas, between January 1, 2005, and December 31, 2010. Visits coded with 1 or more ICD-9 codes for poisoning (965, 977, and their subcodes and supplemental E-codes E850, E858, E935, E947, and E950 and their subcodes) were identified from an administrative database, and further review of the medical records was conducted to identify true cases of acetaminophen exposure or overdose. We then examined the sensitivity, positive predictive value, and percentage of false positives identified by various codes and code combinations to establish which codes most accurately identified acetaminophen exposure or overdose.

**Results:**

Of 1,215 ED visits documented with 1 or more of the selected codes, 316 (26.0%) were a result of acetaminophen exposure or overdose. Sensitivity was highest (87.0%) for the combination of codes 965.4 (poisoning by aromatic analgesics, not elsewhere classified) and E950.0 (suicide and self-inflicted poisoning by analgesics, antipyretics, and antirheumatics), with a positive predictive value of 86.2%. Code 965.4 alone yielded a sensitivity of 85.1%, with a positive predictive value of 92.8%. Code performance varied among age groups and depending on the type of exposure (intentional or unintentional).

**Conclusion:**

ICD-9 codes are useful for ascertaining which ED visits are a result of acetaminophen exposure or overdose within the pediatric population. However, because ICD-9 coding differs by age group and depending on the type of exposure, hypothesis-driven strategies must be utilized for each pediatric age group to avoid misclassification.

## Background

Acetaminophen is the leading pharmaceutical product consumed in the United States [1]. High use rates raise concern for accidental exposure and overdose in children. The Centers for Disease Control and Prevention Morbidity and Mortality Weekly Report noted that more than 50,000 emergency department (ED) visits between 2001 and 2003 were for children younger than 4 years who were treated for a potential pharmaceutical overdose, and most of these children were aged 1 to 2 years. Despite minimization efforts, ED visits for unintentional medication exposures persist, and many of these unintentional exposures involve acetaminophen [2].

Reported rates of acetaminophen-related ED visits among children are commonly extrapolated from the National Electronic Injury Surveillance System–All Injury Program (NEISS-AIP), which contains information on all injuries treated in the ED at a sample of hospitals in the United States. The Centers for Disease Control also collaborates on the NEISS Cooperative Adverse Event Surveillance project (NEISS-CADES) to identify all adverse drug events leading to ED visits for children and teens [3]. According to these data, of all drugs associated with pediatric overdose resulting in ED visits in the United States between 2004 and 2005, acetaminophen accounted for the highest number of cases of unsupervised ingestion (10.5% of cases) and the second highest number of cases of misuse (13.5% of cases) [4]. In addition to these estimates, acetaminophen unintentional pharmaceutical exposure and overdose in children younger than 6 years is collected from the US Poison Control Center as a comparator [5]. However, this information is voluntarily provided to the call center and thus may underestimate the rate of pediatric acetaminophen exposure and overdose because less severe cases may not require a call. Moreover, the information from the American Association of Poison Control Centers National Poison Data System (AAOCC-NPDS) does not include medical examinations or final diagnoses. Estimates of pediatric acetaminophen exposure and overdose are similar between NEISS and AAPCC however, both may be underestimates of the rate of accidental pharmaceutical exposure and overdose among children [6].

Pediatric exposure to and overdose from acetaminophen has received attention from the US Food and Drug Administration Center for Drug Evaluation and Research, which has discussed changing dosing information for over-the-counter products containing acetaminophen for children younger than 12 years [7]. At the 2011 US Food and Drug Administration nonprescription advisory committee meeting, information was presented from the Nationwide Inpatient Sample for 1998–2008, representing 95% of all US hospitalizations during that period. (The Nationwide Inpatient Sample is part of the Healthcare Cost and Utilization Project, which is sponsored by the Agency for Healthcare Research and Quality [7]). These data were collecting using an International Classification of Disease (ICD-9) approach. Two primary codes were used to identify cases of acetaminophen exposure or overdose among children: ICD-9 codes 965.4 (poisoning by aromatic analgesics, not elsewhere classified [NEC]) and E850.4 (accidental poisoning by aromatic analgesics, NEC) [7]. Clinical records were not available for review, and it was assumed that all cases identified were acetaminophen exposures or overdoses. This is one of few instances in which ICD-9 codes were explicitly used to identify discharges related to acetaminophen exposure among children. As no “gold standard” measures exist and current surveillance and reporting methods may be flawed, it is important to re-evaluate and assess novel approaches for estimating acetaminophen overdose among the pediatric population [6].

The objective of this study was to evaluate the utility of various ICD-9 codes or combinations of codes for identifying ED visits resulting from acetaminophen exposure or overdose among children.

## Methods

### Data collection

We collected records of ED visits related to acetaminophen exposure or overdose between the dates of January 1, 2005, and December 31, 2010, at Texas Children’s Hospital in Houston, Texas, which has the largest pediatric ED in south-central Texas, admitting approximately 85,000 patients each year. Administrative records of the ED visits documented diagnostic information using specific ICD-9 codes (Table 1) [8]. We selected records containing codes related to acetaminophen and analgesic exposure, specifically codes 965 (poisoning by analgesics, antipyretics, and antirheumatics), 977 (poisoning by other and unspecified drugs and medicinal substances), or any of the subcodes of 965 or 977. In addition, we selected records containing certain supplemental E-codes or any of their subcodes: E850 (accidental poisoning by drugs, medicinal substances, and biological substances), E858 (accidental poisoning by other drugs), E935 (aromatic analgesics, NEC, causing adverse effects in therapeutic use), E947 (other and unspecified drugs and medicinal substances causing adverse effects in therapeutic use), and E950 (suicide and self-inflicted poisoning by solid or liquid substances). Although the coding algorithms are defined by ‘poisoning’ many cases reviewed did not include poisoning. We therefore refer to these cases as acetaminophen-related exposures or overdose. Our search included all diagnostic codes used to document each visit, which at Texas Children’s Hospital can number up to 25. Demographic information was collected from the medical record. The University of Texas MD Anderson Cancer Center and Baylor College of Medicine Institutional Review Boards approved this study protocol.

**Table 1:** International Classification of Disease (ICD-9) codes and supplemental E-codes used to identify emergency department visits resulting from pediatric acetaminophen exposure or overdose*

**ICD-9 code**	**Description**
Regular codes	
965.0	Poisoning by opium (alkaloids), unspecified
965.01	Poisoning by heroin
965.02	Poisoning by methadone
965.09	Poisoning by other opiates and related narcotics
965.1	Poisoning by salicylates
**965.4**	**Poisoning by aromatic analgesics, not elsewhere classified**
965.5	Poisoning by pyrazole derivatives
965.61	Poisoning by propionic acid derivatives
965.69	Poisoning by other antirheumatics
965.7	Poisoning by other nonnarcotic analgesics
965.8	Poisoning by other specified analgesics and antipyretics
965.9	Poisoning by unspecified analgesics and antipyretics
977.0	Poisoning by dietetics
977.1	Poisoning by lipotropic drugs
977.2	Poisoning by antidotes and chelating agents, not elsewhere classified
977.3	Poisoning by alcohol deterrents
977.4	Poisoning by pharmaceutical excipients
977.8	Poisoning by other specified drugs and medicinal substances
977.9	Poisoning by unspecified drugs or medicinal substances
Supplemental codes	
E850.0	Accidental poisoning by heroin
E850.1	Accidental poisoning by methadone
E850.2	Accidental poisoning by other opiates and related narcotics
**E850.3**	**Accidental poisoning by salicylates**
**E850.4**	**Accidental poisoning by aromatic analgesics, not elsewhere classified**
E850.5	Accidental poisoning by pyrazole derivatives
E850.6	Accidental poisoning by antirheumatics (antiphlogistics)
**E850.7**	**Accidental poisoning by other nonnarcotic analgesics**
E850.8	Accidental poisoning by other specified analgesics and antipyretics
E850.9	Accidental poisoning by unspecified analgesics or antipyretics
E858.0	Accidental poisoning by hormones and synthetic substitutes
E858.1	Accidental poisoning by primarily systemic agents
E858.2	Accidental poisoning by agents primarily affecting blood constituents
E858.3	Accidental poisoning by agents primarily affecting cardiovascular system
E858.4	Accidental poisoning by agents primarily affecting gastrointestinal system
E858.5	Accidental poisoning by water, mineral, and uric acid metabolism drugs
E858.6	Accidental poisoning by agents primarily acting on the smooth and skeletal muscles and respiratory system
E858.7	Accidental poisoning by agents primarily affecting skin and mucous membranes; ophthalmological, otorhinolaryngological, and dental drugs
E858.8	Accidental poisoning by other specified drugs
E858.9	Accidental poisoning by unspecified drugs
E935.0	Heroin causing adverse effects in therapeutic use
E935.1	Methadone causing adverse effects in therapeutic use
E935.2	Other opiates and related narcotics causing adverse effects in therapeutic use
E935.3	Salicylates causing adverse effects in therapeutic use
E935.4	Aromatic analgesics, not elsewhere classified, causing adverse effects in therapeutic use
E935.5	Pyrazole derivatives causing adverse effects in therapeutic use
E935.6	Antirheumatics [antiphlogistics] causing adverse effects in therapeutic use
E935.7	Other nonnarcotic analgesics causing adverse effects in therapeutic use
E935.8	Other specified analgesics and antipyretics causing adverse effects in therapeutic use
E935.9	Unspecified analgesics and antipyretics causing adverse effects in therapeutic use
**E950.0**	**Suicide and self-inflicted poisoning by analgesics, antipyretics, and antirheumatics**
E950.5	Suicide and self-inflicted poisoning by unspecified drugs or medicinal substances

*Boldface type indicates codes that achieved the *a priori* set threshold of a positive predictive value ≥ 50%.

### Chart review

A detailed review of the selected medical records was conducted by trained research assistants to confirm that the coding accurately reflected cases of pediatric acetaminophen exposure or overdose. Each record of the visit date and consecutive hospital stay, if applicable, was reviewed by a research assistant. A standardized data abstraction worksheet with a detailed codebook was used for each visit. We defined cases as those in which a suspected or known accidental ingestion or an overdose of an acetaminophen-containing product were clearly documented. We included suspected ingestions because often a child was found with an open bottle but the caregiver was not certain whether the child had ingested any of the contents. The type of acetaminophen exposure or overdose was categorized as unintentional, intentional, or illicit unintended use. Unintentional acetaminophen exposure occurred in cases in which the caregiver suspected ingestion, a provider gave the child an incorrect dose, or the child ingested acetaminophen without an adult’s supervision or intention to treat. Intentional acetaminophen exposure occurred in cases in which the patient admitted to intentionally ingesting the medication with the purpose of harming him or herself, or if the treating physician or social worker note included the key words ‘intentional’ or ‘suicidal use.’ Illicit unintended use of acetaminophen occurred in cases in which the medical record notes documented the patient admitting to intentionally ingesting the medication for the purpose of “getting high.” All charts reviewed with information regarding the visit fell into one of the previously defined categories. Uncertainties regarding intention category were reviewed with a second chart review and consensus among two reviewers. Visits identified by the codes that were missing medical records or did not have any notes regarding the visit were excluded.

### Statistical analysis

We estimated the sensitivity for each ICD-9 code of interest under the assumption that all possible acetaminophen-related ED visits had been coded by at least 1 of the codes of interest, implying 100% capture with the codes listed in Table 1. Sensitivity of a code was estimated as the percentage of all cases of acetaminophen exposure or overdose identified by the code. We also calculated the positive predictive value (PPV) for each code of interest as the percentage of acetaminophen-related visits among all ED visits identified by that code. In addition, the percentage of ED visits identified by each code that were later determined not to be cases of acetaminophen exposure or overdose was also calculated, determining in the percentage of false-positive cases identified by each code. We report false positive cases and specificity interchangeably as specificity is equal to one minus false positives. The performance of combinations of codes was also evaluated using sensitivity, PPV, and percentage of false positives. These results (for both single codes and combinations of codes) were then stratified by age categories that are based on previous research related to pediatric acetaminophen-related ED visits: all ages, less than 6 years, ages 6–14 years, and 15 years or greater [9]. Trends in overdose were compared for all years. For the final analysis, we included only codes used for 1 or more visits that had a PPV of at least 50% to avoid inclusion of codes with a high proportion of false positives. Codes included in the final analysis are shown in boldface type in Table 1. PPV and sensitivity were calculated for these codes within each age group. Data were analyzed using Stata 10.0 software (StataCorp, College Station, TX).

## Results

### Identification of acetaminophen-related ED visits

During the specified timeframe, there were 478,973 admissions to the ED of Texas Children’s Hospital, and 1,215 (0.25%) of these visits were documented with at least 1 of the relevant ICD-9 codes shown in Table 1. Of these 1,215 visits (for 1,119 unique patients), 316 visits (26.0%) were considered to be for cases of potential acetaminophen exposure or overdose after review of the records. Eight hundred ninety-nine visits were not related to acetaminophen exposure or overdose; we categorized them broadly into 1 of 3 groups: (1) visits for drug adverse events without any mention or suspicion of overdose: 40 visits (4.4%); (2) visits for potential exposure to or overdose from a drug other than acetaminophen: 569 visits (63.3%); and (3) visits that did not appear to be related to drug overdose (in some cases acetaminophen may have been in the medication list, but without reference to intoxication or suspicion of overdose): 290 cases (32.3%). The median age of patients in the 316 relevant visits was 9.8 years (range 3.6 months to 20.2 years) and 61.7% were female (Table 2).

**Table 2:** Characteristics of pediatric patients visiting the emergency department for acetaminophen exposure or overdose (n = 316 visits)

**Variable**	**No.**	** %**
Age*		
0-5 years	154	48.7
6-14 years	71	22.5
15-24 years	91	28.8
Female	195	61.7
Ethnicity		
White	133	42.1
Hispanic	103	32.6
Black	52	16.4
Asian	11	3.5
Other/unknown	17	5.4

*Median age for all visits: 9 years (range, 3.6 months to 21 years).

### ICD-9 code performance

General codes among those selected for this study that did not achieve the *a priori* set threshold of PPV ≥ 50% included 977 (poisoning by other and unspecified drugs and medicinal substances) and all subcodes of the following supplemental E-codes: E858 (accidental poisoning by other drugs), E935 (aromatic analgesics, not elsewhere classified, causing adverse effects in therapeutic use), and E947 (other and unspecified drugs and medicinal substances causing adverse effects in therapeutic use).

Table 3 shows the sensitivity, PPV, and percentage of false positives for each code and each code combination with a PPV of at least 50%, by age group. With all ages considered together, the highest sensitivity, 87.0%, was observed for the combination of codes 965.4 (poisoning by aromatic analgesic, NEC) or E950 (suicide and self-inflicted poisoning by analgesics, antipyretics, and antirheumatics), with a PPV of 86.2%. The sensitivity for code E850.4 (accidental poisoning by aromatic analgesics, NEC) in all age groups was low (25.9%), but only 5.7% of the visits documented with this code were false positives. Only 1 visit was coded with 850.7 (accidental poisoning by other non-narcotic analgesics), and therefore the PPV for this code was 100%. Code 965.4 had a sensitivity of 85.1% and a PPV of 92.8%. Of note, all cases of acetaminophen exposure or overdose identified by code E850.4 were also identified by code 965.4. In addition, the majority (91.5%) of cases identified by code E950.0 were also coded as 965.4.

**Table 3:** Sensitivity, positive predictive value (PPV), and percentage of false positives identified by selected International Classification of Disease (ICD-9) codes* used to document emergency department (ED) visits for acetaminophen exposure or overdose, stratified by age group

**Age category (Years) (Number of cases)**	**ICD-9 code**	**Total number of visits**	**Total number of cases**	**Sensitivity (%)**	**PPV (%)**	**False positive visits (%)**
	965.4	290	269	85.1%	92.8%	7.2%
	E850.4	87	82	25.9%	94.3%	5.7%
	E850.7	1	1	0.3%	100.0%	0.0%
All ages	E950.0	98	71	22.5%	72.4%	27.6%
(N=316)	965.4 OR E850.4	290	269	85.1%	92.8%	7.2%
	965.4 OR E950.0	319	275	87.0%	86.2%	13.8%
	E850.4 OR E950.0	185	153	48.4%	82.7%	17.3%
	965.4, E850.4 OR E950.0	319	275	87.0%	86.2%	13.8%
Age 0-5	965.4	140	133	86.4%	95.0%	5.0%
	E850.4	75	72	46.8%	96.0%	4.0%
(N=154)	965.4 OR E850.4	140	133	86.4%	95.0%	5.0%
	965.4	62	57	80.3%	91.9%	8.1%
	E850.3	3	2	2.8%	66.7%	33.3%
Age 6-14	E850.4	9	7	9.9%	77.8%	22.2%
(N=71)	E950.0	42	29	40.8%	69.0%	31.0%
	965.4 OR E850.3	65	59	83.1%	90.8%	9.2%
	965.4 OR E850.4	62	57	80.3%	91.9%	8.1%
	965.4 OR E950.0	78	61	85.9%	78.2%	21.8%
	E850.3 OR E950.0	45	31	43.7%	68.9%	31.1%
	E850.4 OR E950.0	51	36	50.7%	70.6%	29.4%
	965.4, E850.3 OR E850.4	65	59	83.1%	90.8%	9.2%
	965.4, E850.3 OR E950.0	81	63	88.7%	77.8%	22.2%
	965.4, E850.4 OR E950.0	78	61	85.9%	78.2%	21.8%
	E850.3 OR E850.4 OR E950.0	54	38	53.5%	70.4%	29.6%
	965.4, E850.3, E850.4 OR E950.0	81	63	88.7%	77.8%	22.2%
	965.4	88	79	86.8%	89.8%	10.2%
Age 15-24	E850.4	3	3	3.3%	100.0%	0.0%
(N=91)	E950.0	56	42	46.2%	75.0%	25.0%
	965.4 OR E850.4	88	79	86.8%	89.8%	10.2%
	965.4 OR E950.0	101	81	89.0%	80.2%	19.8%
	E850.4 OR E950.0	59	45	49.5%	76.3%	23.7%
	965.4, E850.4 OR E950.0	101	81	89.0%	80.2%	19.8%

*ICD-9 codes: 965.4: poisoning by aromatic analgesics, not elsewhere classified; E850.3: accidental poisoning by salicylates; E850.4: accidental poisoning by aromatic analgesics, not elsewhere classified; E850.7: accidental poisoning by other nonnarcotic analgesics; E950.0: suicide and self-inflicted poisoning by analgesics, antipyretics, and antirheumatics.

When we included only the codes with a PPV of at least 50% in our analysis, sensitivity was high. However, 41 (13.0%) of the 316 cases of acetaminophen exposure or overdose were not identified by these codes. These cases were identified by codes 965.09 (poisoning by other opiates and related narcotics; 31.7%), 965.1 (poisoning by salicylates; 12.2%), or 977.8 (poisoning by other specified drugs and medicinal substances; 9.8%); 13 additional codes identified fewer than 3 cases each.

Several differences in code performance were noted after stratification by age group (Table 3). The youngest group (0–5 years) had the highest number of visits identified by the specified codes. For age group of 6–14 years, 80.3% of the cases were identified by 965.4, with a PPV of 91.9%. The code for accidental poisoning by aromatic analgesics (E850.4) had the second highest PPV (77.8%), but the sensitivity was very low (9.9%). Within this age group, code E950.0 (suicide and self-inflicted poisoning by analgesics, antipyretics, and antirheumatics) identified a considerable proportion of cases, resulting in a sensitivity of 40.8% and a relatively high PPV (69.0%). For the oldest group (ages 15–24), code 965.4 identified 86.8% of cases, with a 10.2% false positive rate. Combining codes 965.4 and E950.0 increased sensitivity to 89.0% but also increased the percentage of false positives to 19.8%.

Table 4 shows the types of acetaminophen exposure or overdose that occurred in cases that were verified by medical record review. Among all age groups, of the 82 cases identified by code E850.4 (indicative of an accidental poisoning) 75 were unintentional acetaminophen exposure or overdose and 6 were intentional. Of the 71 cases identified by E950.0, describing suicide or self-inflicted poisoning, 1 case was unintentional and 70 (98.6%; 95% CI 92.4%, 100%) were intentional. In the youngest age group (0–5 years), all cases who were coded E850.4 (indicative of an accidental poisoning) were unintentional. It is important to note, however, in this age group all cases were unintentional; after chart review there were no intentional cases identified, however the more specific code E850.4 was only used in 72 of the 133 cases (54.1%). In the age group of 6–14 years, of the 29 cases that were coded E950.0 all 29 were intentional. Finally, for the oldest age group (15–24 years), of the 42 cases coded E950.0, which indicates suicide or self-inflicted poisoning, 41 (97.6%) of them were intentional.

**Table 4:** Type of acetaminophen exposure or overdose that occurred with each International Classification of Disease (ICD-9) code used to document the emergency department (ED) visit, by age group

**Age category**	**Description**	**ICD-9 code**	**Visits**	**Cases**	**Unintentional, N (%; 95% CI)**	**Intentional, N (%; 95% CI)**	**Illicit unintended use, N (%; 95% CI)**
**Total**			**N=1,215**	**N=316**	**N=172**	**N=139**	**N=5**
	Poisoning	965.4	290	269	146 (54.3; 48.1-60.3)	119 (44.2; 38.2-50.4)	4 (1.5; 0.4-3.8)
All ages	**Accidental**	**E850.4**	87	82	**75 (91.5; 83.2-96.5)**	6 (7.3; 2.7-15.2)	1 (1.2; 0.0-6.6)
(N=316)	**Accidental**	**E850.7**	1	1	**1 (100.0; 2.5-100.0)**	0 (0.0)	0 (0)
	**Suicide/self-inflicted**	**E950.0**	98	71	1 (1.4; 0.0-7.6)	**70 (98.6; 92.4-100.0)**	0 (0)
Age 0-5	Poisoning	965.4	140	133	133 (100; 97.3-100.0)	0 (0)	0 (0)
(N=154)	**Accidental**	**E850.4**	75	72	**72 (100; 95.0-100.0)**	0 (0)	0 (0)
	Poisoning	965.4	62	57	8 (14.0; 6.3-25.8)	47 (82.5; 70.1-91.3)	2 (3.5; 0.4-12.1)
Age 6-14	**Accidental**	E850.3	3	2	**1 (50.0; 1.3-98.7)**	1 (50.0; 1.3-98.7)	0 (0)
(N=71)	**Accidental**	E850.4	9	7	**3 (42.9; 9.9-81.6)**	4 (57.1; 18.4-90.1)	0 (0)
	**Suicide/self-inflicted**	E950.0	42	29	0 (0)	**29 (100; 88.1-100.0)**	0 (0)
Age 15-24	Poisoning	965.4	88	79	5 (6.3; 2.1-14.2)	72 (91.1; 82.6-96.4)	2 (2.5; 0.3-8.8)
(N=91)	**Accidental**	**E850.4**	3	3	**0 (0)**	2 (66.7; 9.4; 99.2)	1 (33.3; 0.8-90.6)
	**Suicide/self-inflicted**	E950.0	56	42	1 (2.4; 0.1-12.6)	**41 (97.6; 87.4-99.9)**	0 (0)

*CI* confidence intervals; Boldface type indicates codes who’s codes correctly identified intention type.

Stratification by year (2005 to 2010) indicated a decreasing number of ED visits identified by 1 or more of the ICD-9 codes of interest. However, the number of cases of acetaminophen exposure or overdose, according to the medical record review, did not change significantly over the period studied, suggesting that coding procedures became more efficient over time.

## Discussion and conclusion

We selected records of ED visits using selected ICD-9 codes, and from these, cases in which acetaminophen exposure or overdose was suspected or known were identified after a thorough review of medical records. Review of the medical records used in our study showed that 316 out of the 1,215 ED visits identified by the ICD-9 codes of interest (26.0%) were related to acetaminophen exposure or overdose, which would result in a 3:1 ratio of false positive cases. However, when we included in our analysis only the codes with a PPV of at least 50% to avoid a large number of false positives, we captured only 87.0% of cases of acetaminophen exposure or overdose. This suggests that different strategies might be appropriate in different situations when using ICD-9 codes to identify rates of acetaminophen exposure or overdose in pediatric patients. In our analysis, the most cases of acetaminophen exposure or overdose for all ages considered together were identified using the combination of codes 965.4 (poisoning by aromatic analgesic, NEC) or E950.0 (suicide and self-inflicted poisoning by analgesics, antipyretics, and antirheumatics), with a sensitivity of 87.0%, although this combination of codes also yielded a 13.8% false positive rate. For children less than 6 years old, code 965.4 captured 86.4% of cases, with a very low proportion of false positives (5.0%). It is important to recognize that although some strategies are efficient in capturing the most cases, these strategies may also result in a significant number of misclassified cases. It is also important to note all cases coded with 965.4 were also coded E850.4, indicating accidental poisoning. For this reason, using the combination logic of code 965.4 in addition to E850.4 and E950.0 resulted in the same sensitivity as using 965.4 and E950.0 alone.

On the other hand, some studies may require high specificity, such as case–control studies in which certainty about the relevance of the selected cases is important. Under these circumstances, the best code to use, according to our analysis, was 965.4, which resulted in a 7.2% false positive rate for all ages combined (5.0% for 0–5 years, 8.1% for 6–14 years, and 10.2% for 15–24 years); and sensitivity was 85.1% overall (86.4% for 0–5 years, 80.3% for 8–14 years, and 86.8% for 15–24 years).

Our analysis showed that ICD-9 codes could be used to identify circumstantial subgroups of pediatric patients exposed to acetaminophen. For example, for identifying ED visits for young children unintentionally exposed to acetaminophen, using both code 965.4 and accidental codes, such as E850.4 and/or E850.7, maximized sensitivity and PPV. Similarly, for identifying ED visits related to intentional acetaminophen exposure, using codes 950.0 (for suicide or self-inflicted poisoning) and 965.4 in the search led to the most precise identification of cases of interest. To the best of our knowledge, this is the first study to examine the validity of ICD-9 codes used in an ED administrative database to identify potential exposure to or overdose of acetaminophen products in a pediatric population.

ICD-9 codes are useful for ascertaining which ED visits is a result of acetaminophen exposure or overdose within the pediatric population. Previous studies have used ICD-9 codes to evaluate national trends in admissions for acetaminophen exposure in adults [10]. The National Hospital Ambulatory Care Survey, National Hospital Discharge Survey, and the Centers for Disease Control’s database of mortality all used ICD-9 code 965.4 to identify ED visits resulting from acetaminophen poisoning [11-13]. They also used code E850.4 to identify ED visits resulting from accidental poisoning by aromatic analgesics, NEC and code E850.2 to identify accidental poisoning by other opiates and related narcotics. In addition, ICD-9 codes were used to determine the type of acetaminophen exposure (intentional or unintentional), particularly in the National Hospital Discharge Survey and in national studies examining causes of death.

In our study, the codes used for cases of unintentional acetaminophen exposure differed from those used for cases of intentional acetaminophen exposure or overdose among our oldest group of patients (15–24 years). Code E950.0 (suicide and self-inflicted poisoning by analgesics, antipyretics, and antirheumatics) had a 75.0% PPV, although this code alone was not capable of capturing the majority of cases. These findings are consistent with data from others with respect to age and rates of intentional overdose [9]. Budnitz et al. identified no cases of intentional overdose among children younger than 6 years between 2006 and 2007 using data from the NEISS, but older patients (ages 15–24 years) had a higher rate of emergency department visits for intentional overdoses (over 45 per 100,000 individuals per year).

We found that the number of ED admissions documented with the codes we selected peaked in 2005 and 2007 and sharply decreased between 2008 and 2010. However, the number of confirmed cases per year, was consistent throughout the period studied, indicating that misleading trends may result from the use of incorrect diagnosis codes. From 2007 to 2010, code 965.4 was capable of capturing 100% of cases, leaving no false positives. Changes in coding, policy, or public awareness may have played a part in the decreased number of ED admissions documented with our selected codes during 2007–2010. Previous reports examining data from 2001 to 2008 showed an increase in admissions, ED visits, and calls related to ingestions of pharmaceutical products. Using the National Poison Data System, Bond et al. reported a 57% increase in cases of unintentional acetaminophen exposure or overdose and a 71% increase in cases of therapeutic error nationally between 2001 and 2008 [5]. These estimates may not be comparable to those in our study because medications that include acetaminophen were placed in a separate drug group from those containing acetaminophen only in the analysis.

Several limitations should be considered when interpreting our data. First, our findings are representative of results from a single pediatric hospital within a specified time range. Although we examined data from the largest pediatric hospital in Texas, data from other hospitals serving different areas may differ in coding practices, and coding algorithms should be validated in additional hospitals over alternative time periods. In addition, we assumed that all visits related to acetaminophen exposure or overdose were identified by at least 1 of the codes we selected. Due to the large amount of visits, only one trained research assistant reviewed the medical records and concordance statistics are not available, however, a standardized consensus approach was used to classify these cases. Not all true acetaminophen exposures/overdoses might have been captured as some may not have been assigned our selected codes, but if any, we believe this proportion would be very small given our broad code selection, including several general codes with low sensitivity and specificity. In interpreting our findings it is important to note distinctions between exposure and overdose. Since in most occasions ingestion was not observed, overdose could not be confirmed. While the clinical implications of these differences are important, the purpose of our study was to evaluate the value of diagnostic algorithms to identify a majority of potential overdoses. Finally, we examined only the ICD-9-CM coding system, which may not be comparable with the ICD-10-CM coding system for future studies.

Despite efforts to reduce unintentional medication exposures among children, overdose of acetaminophen in this population continues to be a public health concern. Development of prevention strategies and educational activities to reduce pediatric exposure to or overdose of acetaminophen depends on reliable data regarding trends in acetaminophen-related ED visits. Data regarding ED visits related to acetaminophen exposure or overdose is sparse, and detailed abstraction of medical records can be time-consuming and costly. Using reliable ICD-9 codes or combinations of codes can be a useful strategy to estimate the number of acetaminophen exposures and overdoses in the pediatric population. However, researchers and users must be cognizant that approaches maximizing case identification will also result in a significant proportion of false positive cases, whereas strategies to identify only true positive cases will result in an underestimate of the true burden of acetaminophen exposure.

## Competing interests

The authors declare that they have no competing interests.

## Authors’ contributions

SA, MES-A, and CF conceived of and designed the study. SA and MES-A acquired the data and SA, SP, and MES-A analyzed and interpreted the data. SA drafted the manuscript and CF, SP, and MES-A provided critical revision of the manuscript for content. SA and SP performed statistical analysis. MES-A obtained funding. SA, CF, and MES-A provided administrative, technical, and material support. MES-A supervised the study. All authors read and approved the final manuscript.

## Pre-publication history

The pre-publication history for this paper can be accessed here:

http://www.biomedcentral.com/1472-6963/13/72/prepub
